# Author Response: Letter to the Editor—Reconsidering Control Logic and Translational Interpretation in TNEA‐Based Modulation of Hippocampal Oscillations

**DOI:** 10.1002/advs.77636

**Published:** 2026-09-08

**Authors:** Zhongzhao Guo, Deheng Wang

**Affiliations:** ^1^ School of Integrative Medicine Shanghai University of Traditional Chinese Medicine Shanghai China

We thank the correspondents for their careful reading and thoughtful comments on our study [1]. Below, we respond to each point and clarify the methodological rationale and interpretive framework of our work.

## 1 Non‐Acupoint Control

Nevertheless, behavioral data indicate that this non‐acupoint control group did not produce cognitive improvement. This functional dissociation at the behavioral level indicates that, despite the small cranial dimensions of mice, the millimeter‐scale displacement was enough to engage distinct neural substrates. We acknowledge that millimeter‐scale spatial separation on the mouse scalp does not guarantee complete neural isolation, and we do not rely on this assumption alone. Importantly, the electrophysiological assessments employed a separate EA sham control group (needling without electrical stimulation) that provides a more rigorous dissection of the effects specifically attributable to electrical neuromodulation, independent of the non‐acupoint spatial comparison. Although the 1‐mm separation for the non‐acupoint control did not produce acupuncture‐like behavioral effects in our study, we acknowledge that this spatial scale may not entirely eliminate local diffusion or nonspecific mechanical effects. Future studies should incorporate nerve block or lesion controls where feasible.

## 2 Repeated Needle Insertion

Repeated needle insertions are routinely performed in long‐term acupuncture protocols, in both rodent models and humans, and are typically associated with only minimal, transient tissue injury that does not reach clinical significance [2]. The 20‐session treatment over 4 weeks allows for adequate inter‐session recovery. More importantly, both the treatment and control groups underwent identical needling procedures, differing only in stimulation location or electrical activation. Any nonspecific effects related to repeated needling, including inflammatory or neuroimmune responses, would therefore be equally distributed across groups and cannot explain the observed differences in cognitive performance or hippocampal oscillatory activity.

## 3 Mechanistic Consistency of PV^+^

We interpret this finding differently, not as an inconsistency, but as a central insight of the study. It reveals that pathological improvement (reduced amyloid deposition and microglial activation) is necessary but not sufficient for cognitive recovery, which PV^+^ interneuron activity and network oscillations are required for these pathological changes to be translated into functional improvement [3]. The suggestion that PV^+^ interneurons serve only a permissive role is not supported by the experimental evidence. Chemogenetic inhibition reversed cognitive improvements that had already been established by TNEA treatment. This demonstrates that PV^+^ interneuron activity is functionally required, not merely correlated or permissively associated, for the behavioral effects. Biological mechanisms frequently operate across multiple hierarchical levels, from pathology and neuroinflammation to interneuron function, network oscillations, and behavior. Dissecting one critical pathway does not imply exclusion of other contributing mechanisms.

## 4 Causal Directionality of Oscillatory

Sensitivity to arousal, novelty, and movement is an inherent property of many neurophysiological signals, including LFP, neuronal spikes, and calcium imaging. These potential confounds are typically minimized through standardized behavioral protocols, habituation procedures, and repeated recordings, all of which were incorporated into this study. We acknowledge that hippocampal oscillations are emergent network phenomena and that establishing causal directionality requires careful experimental design. We appreciate the correspondents’ suggestion that direct, independent manipulation of oscillatory dynamics, separate from both acupuncture and their upstream generating circuits, would represent an ideal approach to testing causality. Although this is theoretically attractive, achieving such selective manipulation independently of the generating circuits is not currently feasible, and such isolation is not routinely required for mechanistic inference in systems neuroscience. Instead, our study established causality through chemogenetic manipulation of PV^+^ interneurons, which produced the predicted changes in both hippocampal oscillations and behavioral outcomes [4, 5]. This approach follows the established paradigm for causal neural circuit dissection in behaving animals and defines a causal chain from PV^+^ interneuron activity to oscillatory dynamics and cognitive function.

## 5 Translational Implications

We thank the correspondents for raising the important issue of translational interpretation. We acknowledge that the non‐acupoint spatial comparison could be read as implying millimeter‐level precision. However, the central experimental finding of our study is the comparison between EA and EA sham, which isolates the role of electrical stimulation parameters. We also wish to clarify a factual point: the EA sham control group involved needle insertion without manual manipulation or electrical stimulation — it is not, as the correspondents suggest “repeated manual acupuncture”. The core experimental comparison was therefore between needling with versus without electrical stimulation (EA vs EA sham), designed to isolate the effects of electrical stimulation parameters. We fully agree that bridging rodent scalp stimulation parameters to human clinical protocols requires careful consideration and additional investigation, and we have explicitly acknowledged this limitation in the original article.

We appreciate the opportunity to clarify these points and believe that the methodological controls, chemogenetic validation, and standardized assessments incorporated in our study adequately address the concerns raised. The hierarchical causal model supported by our data, which links TNEA to pathology reduction, PV^+^ interneuron restoration, oscillatory normalization, and cognitive rescue, provides a coherent framework for interpreting the findings. The available evidence therefore supports the interpretation that the reported findings represent a meaningful mechanistic contribution.

## Conflicts of Interest

The authors declare no conflicts of interest.

## Data Availability

The data that support the findings of this study are available from the corresponding author upon reasonable request.

## References

[1] Z. Guo , H. Ni , Y. Lu , et al., “TNEA Regulates Hippocampal Oscillation by Improving Inhibitory Synaptic Plasticity to Ameliorates Cognitive Impairment in Alzheimer's Disease,” Advanced Science 13, no. 3 (2026): 10885, 10.1002/advs.202510885. PMC12806385 41215663

[2] P. Bäumler , W. Zhang , T. Stübinger , and D. Irnich , “Acupuncture‐Related Adverse Events: Systematic Review and Meta‐Analyses of Prospective Clinical Studies,” BMJ Open 11, no. 9 (2021): 045961, 10.1136/bmjopen-2020-045961. PMC8422480 34489268

[3] S. Hijazi , A. B. Smit , and R. E. van Kesteren , “Fast‐Spiking Parvalbumin‐Positive Interneurons in Brain Physiology and Alzheimer's Disease,” Molecular Psychiatry 28, no. 12 (2023): 4954–4967, 10.1038/s41380-023-02168-y. 37419975 PMC11041664

[4] F. Xia , B. A. Richards , M. M. Tran , S. A. Josselyn , K. Takehara‐Nishiuchi , and P. W. Frankland , “Parvalbumin‐Positive Interneurons Mediate Neocortical‐Hippocampal Interactions that are Necessary for Memory Consolidation,” eLife 6 (2017): 27868, 10.7554/eLife.27868. PMC5655147 28960176

[5] N. Ognjanovski , S. Schaeffer , J. Wu , et al., “Parvalbumin‐Expressing Interneurons Coordinate Hippocampal Network Dynamics Required for Memory Consolidation,” Nature Communications 8, no. 1 (2017): 15039, 10.1038/ncomms15039. PMC5384212 28382952

